# Anxiety and coping strategies during the COVID-19 pandemic: A cross-sectional study of staff and students from a tertiary education center in Malaysia

**DOI:** 10.3389/fpubh.2022.936486

**Published:** 2022-10-06

**Authors:** Kai Wei Lee, Sook Fan Yap, Hooi Tin Ong, Kai Shuen Pheh, Munn Sann Lye

**Affiliations:** ^1^Department of Pre-Clinical Sciences, Faculty of Medicine and Health Sciences, Universiti Tunku Abdul Rahman, Kajang, Selangor, Malaysia; ^2^Centre for Research on Communicable Diseases, Universiti Tunku Abdul Rahman, Kajang, Malaysia; ^3^Department of Psychology and Counselling, Faculty of Arts and Social Science, Universiti Tunku Abdul Rahman, Kajang, Selangor, Malaysia; ^4^Department of Population Medicine, Faculty of Medicine and Health Sciences, Universiti Tunku Abdul Rahman, Kajang, Malaysia

**Keywords:** anxiety, coping, COVID-19, university, Malaysia

## Abstract

**Aim:**

We examined the anxiety levels and coping strategies among staff and students of a tertiary educational institution during the COVID-19 pandemic and determined the association between anxiety level and coping strategies.

**Method:**

Through an online survey, we used Coronavirus Anxiety Scale (CAS) to measure the level of anxiety associated with the COVID-19 crisis and Brief Coping Orientation to Problems Experienced (COPE) to assess the coping responses adopted to handle stressful life events. Coping strategies were classified as adaptive and maladaptive, for which the aggregate sores were calculated. Multiple linear regression was used to determine the predictors of anxiety adjusted for potentially confounding variables. Results from 434 participants were available for analysis.

**Results:**

The mean score (SD) of the CAS was 1.1 (1.8). The mean scores of adaptive and maladaptive coping strategies were 35.69 and 19.28, respectively. Multiple linear regression revealed that maladaptive coping [Adjusted B coefficient = 4.106, *p*-value < 0.001] and presence of comorbidities [Adjusted B coefficient = 1.376, *p*-value = 0.025] significantly predicted anxiety.

**Conclusion:**

Maladaptive coping and presence of comorbidities were the predictors of coronavirus anxiety. The apparent lack of anxiety in relation to COVID-19 and movement restriction is reflective of the reported high level of satisfaction with the support and services provided during the COVID-19 outbreak in Malaysia. Adaptive coping strategies were adopted more frequently than maladaptive. Nevertheless, public education on positive coping strategies and anxiety management may be still be relevant to provide mental health support to address the needs of the general population.

## Introduction

The COVID-19 pandemic is a serious global health problem that poses threats to both the physical and mental wellbeing. A meta-analysis found that the pooled prevalence of anxiety was 31.9% among the general population during the COVID-19 pandemic (1). Another review indicated that anxiety level has increased 3-fold during the COVID-19 outbreak among the general population (2). The anxiety generated could be due to a multitude of reasons including the health risk posed by the virus on themselves and their families, the economic burden on themselves and the fallout at large (3), as well as the enforced social isolation during the pandemic (4). Anxiety is a natural response to stress. The symptoms of anxiety include nervousness, restlessness, fatigue and weakness, palpitations, trouble concentrating and insomnia (5–7). Significantly, studies have suggested that prolonged anxiety can reduce quality of life and weaken the immune system (8, 9) and as a result increases the risk of the SARS-Cov2 infection (10). Further, the restrictive measures imposed to curb the spread of the virus, and the associated changes in lifestyle and work arrangements, as well as the severe limitations in social and physical activities have exacted a heavy toll on people. Over an extended period of time, this could result in mental fatigue and stress. It is not surprising, therefore, that frequency of anxiety has been reported to be an increasing health problem arising out of the pandemic.

The pandemic situation that the world is facing now is not new, albeit, it is much more serious in many aspects compared to outbreaks in the recent past. Likewise, many people affected by previous pandemics have experienced and endured similar situational threats and stress at the individual level. During previous outbreaks, many studies have been carried out to address the issue of mental health and anxiety arising therefrom, and how people handle such problems (11–13). In these studies, it was demonstrated that there is an association between anxiety levels, mental resilience and coping styles (14–16).

With regards to teaching and learning, the seriousness of the current pandemic has led most educational systems to adopt online teaching modes, especially in higher education institutions, with all its challenges to teaching and support staff and to students. Transitioning from traditional face-to-face to online teaching-learning can be an entirely different experience and/or challenge for both the learners and the educators. Regardless, this is a change that they must adapt to within a short time frame in the face of limited alternatives. They are compelled to adopt online platforms that they may or may not prepared for, depending on the expertise and previous exposure to information and communications technology (ICT) (17). It was pointed out by Doucet A, et al. (18) that the readiness of the staff and students to adapt to these changes needs to be assessed to allow for appropriate implementation of supportive measures. In the same paper, it was also emphasized that there is no one uniform pedagogy that can be applied across all online subjects which, understandably, will be quite varied each with its own unique requirements both in terms of presentation and delivery of the subject matter. All these issues could present a significant challenge and stress for the entire university community (17). Lastly, there is the issue of equity in higher education, an example in case is students from economically challenged background who may face problems of affording online learning devices and/or reliable internet services (19). Essentially, both staff and students alike have to adapt to the changes in operational, teaching and learning modes, and at the same time cope with the uncertainties related to the evolution of the SARS-Cov2 virus, the course of the pandemic and thus movement restriction as well as their own infection risk.

As a result, questions on the psychological welfare of members of the teaching community has aroused the interest of the research community, hence, the numerous studies to address this issue. Islam et al. (20) reported that 18.1% (19) and Nayan et al. (21) reported 22% of university students from Bangladesh suffered from serious anxiety. Factors reported to be associated with COVID-19 related anxiety among Bangladeshi university students include lagging academic performance (19) and negative attitudes. A study on Middle-Eastern students from Jordan gave a similar prevalence of 21.5% (22); reported predictors of anxiety were chronic illness and, surprisingly, those with higher income. Yang et al. (23) reported that the prevalence of mild, moderate and severe anxiety among University students in Sichuan Province, China were 31.5, 8.1 and 5.8%, respectively, with medical students and those who paid more attention to pandemic information being more likely to be affected. Another study reported the prevalence of mild, moderate and severe anxiety among medical students in India were 41, 16 and 4%, respectively (24). A local study addressing the impact of COVID-19 related anxiety on mental health among Malaysian university students found that 30.5% experienced mild anxiety, 31.1% moderate anxiety and 26.1% severe anxiety; factors associated with anxiety included age over 20 years, Chinese ethnicity, decrease in family income, spending a lot of time watching COVID-19 related news and lastly, history of personal illness and of SARS-CoV-2 infection among friends and relatives (25).

The next question that would be expected is how people facing the stress and anxiety posed by the pandemic cope, and in what way the coping strategies adopted relates to mental health. A study from the United Kingdom (United Kingdom) found that both adaptive and maladaptive coping strategies were used, including socializing with loved ones, exercising, keeping occupied with work or studies, meditating and keeping positive, avoiding negative news on COVID-19, gaming, and taking alcohol (26). An online survey to examine coping strategies used among netizens, nationalities unspecified, found that a large proportion (68.9%) reported that they just hoped for the best, over half (53.2%), just kept themselves busy while around 30 to 35% used religion, or share their concerns with others (15). Mental disengagement was found to be the most common coping method used by university students in China to handle their anxiety; this was followed by avoidance and seeking social support (27).

With regards to the relationship between coping methods and anxiety, a study among nursing students showed that mental disengagement was predictive of moderate to severe anxiety and lack of humor predictive of severe anxiety (14). Another study done among Polish University students (28), reported that anxiety was significantly and inversely correlated with task-oriented coping style, while anxiety was significantly and positively correlated with emotion-oriented coping style and avoidance-oriented coping style.

From the above, it is apparent that literature on the relationship between coping methods and anxiety in the context of the COVID-19 pandemic is generally lacking, both in numbers and coverage. Hence the attempt of the present study to provide some additional data on coping strategies used and their relationship with anxiety among the UTAR community of staff and students. We propose to determine baseline information on the frequency and level of anxiety in this study population, the predictors of COVID-19 anxiety, the frequency of different coping strategies adopted, and the association, if any, between coping strategy and anxiety. Indeed, to date, there has been only one study on anxiety and associated risk factors in Malaysian students (25), and none that address the question of coping strategies and their association with anxiety. Further, we observed that the WHO questionnaire (29) mentions multiple aspects which are deemed relevant and may have an association with anxiety levels, including compliance with preventive measures, satisfaction with support and resources, frequency of updating COVID-19 news, self-risk perception, preparedness, and perceived self-efficacy, and unwanted behavior. Therefore, in the present study, we also explored these factors which are less often addressed in the literature, in comparison to questions on the associations with knowledge, attitude, and socio-demography.

## Materials and methods

### Participants

A cross-sectional online survey was conducted among staff and students of Universiti Tunku Abdul Rahman (UTAR) City campus which consists of 11,541 members (administrative staff, *n* = 414, academic staff, *n* = 717; students, *n* = 10,410). All administrative and academic staff and students of UTAR in Sungai Long Campus were invited to participate in the survey which was conducted between September 1, 2020 and February 28, 2021. This study was part of a broader study which included other aspects related to the COVID-19 pandemic such as knowledge, behavior, self-risk perception (probability, susceptibility and severity) and self-efficacy (protective and avoidance ability) (30).

Sample size was calculated using G^*^Power 3.1 (Linear multiple regression: fixed model, R^2^ deviation from zero) (31). Assuming partial R^2^ of 0.05 (32), effect size (F square) of 0.0526, power of 0.95 and level of significance 0.05, with 18 predictors a minimum sample size of 250 was needed.

### Ethical clearance and procedure

This study was approved by the UTAR Scientific and Ethical Review Committee (approval number: U/SERC/138/2020). Prior to responding to the survey, each participant was informed about the purpose of study, requested to provide signed informed consent and advised about the right to refuse participation and to withdraw at any time.

### Instruments and scoring method

The Coronavirus Anxiety Scale (CAS) was used to measure the level of anxiety associated with the COVID-19 crisis (33) and the Brief COPE to determine the respondent's primary coping styles (34). The CAS is a 5-items mental health screener with 5-point Likert scale ranging from “Not at all” (score 0), “Rarely to twice or less” (score 1), “Several days” (score 2), “More than 7 days” (score 3), and “Nearly every day” (score 4) over the last 2 weeks. The total score ranges from 0 to 20. A cut-off score of ≥ 9 indicates dysfunctional anxiety (33).

The Brief COPE, an abbreviated version of COPE (Coping Orientation to Problems Experienced) is a 28-item self-report questionnaire designed to assess the coping responses adopted to handle stressful life events (34). It contains 14 subscales with 2 items in each subscale, rated by a four-point Likert scale ranging from “I haven't been doing this at all” (score 1), “I've been doing this a little bit” (score 2), “I've been doing this a medium amount” (score 3), and “I have been doing this a lot” (score 4). The higher the score of each subscale, the greater the likelihood for use of that particular coping strategy by the respondent. The 14 subscales in Brief COPE can be classified as “adaptive” and “maladaptive” coping methods. Adaptive coping strategies comprises the first eight scales which consist of active coping, planning, positive reframing, acceptance, humor, religion, using emotional support and using instrumental support. Maladaptive coping comprises the latter six subscales which include self-distraction, denial, venting, substance use, behavioral disengagement and self-blame (34). The scores for the adaptive and maladaptive coping were calculated individually and totalled for each participant; the respective scores were used separately in multiple regression analysis.

The World Health Organization questionnaire “Monitoring knowledge, risk perceptions, preventive behaviors and trust to inform pandemic outbreak response” (29) was used to determine the (i) prevention - own behavior, (ii) frequency of updating news on COVID-19, (iii) satisfaction with support and resources, (iv) self-risk perception (probability and severity), (v) preparedness and perceived self-efficacy, and (vi) unwanted behavior. Prevention (own behavior) was assessed by 10 questions [Scores based on a 7-point Likert scale, options being “Not at all” (1 mark) to “Very much so” (7 marks). The frequency of updating news on COVID-19 was scored based on a 7-point Likert scale [Options include “Never” (1 mark) to “Several times a day” (7 mark)], and satisfaction with support and resources provided with options being “satisfied” and “not satisfied”. Risk perception was explored using three questions covering probability of contracting the infection, susceptibility to the infection and the severity of the illness if infected. Preparedness and perceived self-efficacy comprised two questions on self-protection ability and disease-avoidance ability. Scoring was based on a 7-point Likert scale. In both cases the scores for the individual items were summed to give an aggregate score for statistical analysis. Lastly, unwanted behavior was interrogated using 6 items [Options include “Does not apply” (score 0), “I don't plan to do that” (score 1), “I plan to do that” (score 2), and “I already did that” (score 3)].

### Statistical analysis

Data analysis was performed by using the IBM SPSS Statistics Version 21.0 for Windows. Participants who failed to respond to any one item included in any scale were excluded from statistical analysis. For descriptive statistics, data are presented either as mean ± standard deviation to describe continuous variables or frequency (percentage) to describe categorical and numerical variables. Simple linear regression was conducted to identify factors associated with the coronavirus anxiety. Variables with *p*-values < 0.25 were selected for further analysis using multiple linear regression to obtain adjusted B coefficients and their standard errors using the enter method. Variables with a *p*-value < 0.05 were considered statistically significant.

Further analysis was performed to determine if compliance with preventive measures, frequency of updating about the COVID-19 pandemic, perceived self-risk, preparedness and perceived self-efficacy, and behavior have mediating effects on the relationship between maladaptive coping and the coronavirus anxiety. This was based on 5000 bootstrap resamples and employed the PROCESS macro in IBM SPSS version 21 (35).

## Results

### Characteristics of respondents

Out of 11,541 UTAR staff and students being approached, 435 accessed the online survey and 434 consented and completed the online survey. The demography of participants which comprise 93 staff members and 341 students is presented in Table 1. The mean age of staff members was 36.6 years (range 19–74 years) and that of students was 21.6 years (range 18–35 years), with females making up 67.7% among staff and 58.4% among students. With regards to the educational level, more than half (55.9%) of staff members had a postgraduate or professional degree and 88.5% of students were pursuing an undergraduate degree. Overall, 88.7% were single and 92.9% reported absence of any comorbidity. In terms of living arrangements, 73.7% resided within red (high risk) zones; just over 95% lived in household of 2 or more people; 35% of the participants lived in households with children and 32.3% in households with elderly people.

**Table 1:** Characteristics of participants.

**Characteristics**	**Category**	**All (*n* = 434)**
Age	Mean ± Standard deviation	24.8 ± 8.5
	Median (Interquartile range)	22 (2.3)
	Minimum age—Maximum age	18–74
Gender *n* (%)	Male	172 (39.6)
	Female	262 (60.4)
Role in tertiary education *n* (%)	Administrative staff	43 (9.9)
	Academic staff	50 (11.5)
	Student	341 (78.6)
Highest education level obtained among staff *n* (%)	Secondary	20 (21.5)
	Diploma/Bachelor degree	21 (22.6)
	Postgraduate/professional degree	52 (55.9)
Level of education pursued among students *n* (%)	Foundation	31 (9.1)
	Undergraduate degree	302 (88.6)
	Postgraduate degree	8 (2.3)
Marital status	Single	385 (88.7)
	Married	47 (10.8)
	Divorced	2 (0.5)
Highest education level completed by respondents	Secondary school	353 (81.3)
	Diploma or Bachelor degree	27 (6.2)
	Postgraduate or professional degree	54 (12.4)
Presence of comorbidities	Without	403 (92.9)
	With	9 (2.1)
	Unsure	22 (5.1)
Zone of residence	Green	29 (6.7)
	Yellow	44 (10.1)
	Red	320 (73.7)
	Unsure	41 (9.4)
Household size	Staying alone	14 (3.2)
	2–4 people	229 (52.8)
	≥5 people	191 (44.0)
Living with children	Yes	152 (35.0)
	No	282 (65.0)
Living with elderly	Yes	140 (32.3)
	No	294 (67.7)

Data are presented in frequency (%).

### Compliance with preventive measures, satisfaction with support, frequency of updating information on COVID-19, risk perception, self-efficacy and behavior

The majority (74%) were compliant with the public health measures recommended, more so among the staff members. Likewise, the large majority (82.5%), were satisfied with the support services provided, staff and students equally so. Just under 30% reported that they update themselves regarding the COVID-19 status very frequently (score 6–7), of whom about 10% do so several times a day (Table 2).

**Table 2:** Compliance with COVID-19 preventive measures, satisfaction with support and resources, frequency of updating about the COVID-19 pandemic, risk perception, self-efficacy, unwanted behavior (*n* = 434).

**Characteristics**	**Category**	**All respondents (*n* = 434)**
Compliance with COVID-19 preventive measures (total score = 7)	Low compliance (score 1–2)	Frequency in number and % ()
[Options—“Not at all” (1 mark) to “Very much so” (7 marks)]	Moderate compliance (Score 3–5)	1 (0.2)
	High compliance (Score 6–7)	112 (25.8)
	Mean ± Standard deviation	321 (74.0)
	Median (Interquartile range)	5.9 ± 1.0
	Range	6 (2)
Satisfaction with support and resources	Not satisfied	02-Jul
	Satisfied	76 (17.5)
Frequency of updating about the status of the COVID-19 pandemic (total score = 7)	Infrequent (score 1–2)	358 (82.5)
[Options—“Never” (1 mark) to “Several times a day” (7 mark)]	Frequent (Score 3–5)	38 (8.8)
	Very frequent (Score 6–7)	274 (63.1)
	Mean ± Standard deviation	122 (28.1)
	Median (Interquartile range)	4.7 ± 1.4
	Range	5 (2)
Self-risk perception (total score = 21)	Mean ± Standard deviation	01-Jul
[Options—“Not at all” (1 mark) to “Very much so” (7 marks)]		11.47 ± 2.66
	Median (Interquartile range)	12.0 (3.0)
	Range	May-21
Preparedness and perceived self-efficacy (total score = 14)	Mean ± Standard deviation	10.25 ± 1.73
[Options—“Not at all” (1 mark) to “Very much so” (7 marks)]	Median (Interquartile range)	10.0 (2.0)
	Range	May-14
Unwanted behavior (total score = 18)	Mean ± Standard deviation	7.21 ± 3.33
[Options—“Does not apply” (score 0), “I don't plan to do that” (score 1), “I plan to do that” (score 2) and “I already did that” (score 3)].	Median (Interquartile range)	7.0 (4.0)
	Range	0–18

### Coping methods

Brief-COPE exhibited good internal consistency in this study (**Appendix 1**); the Cronbach's alpha coefficient for subscales ranged from 0.576 (Planning) to 0.936 (Substance use) which is comparable to that reported (18). The frequency of response for the 28 items of the Brief-COPE are summarized in **Appendix 2**.

For analysis, the Brief COPE items were categorized into adaptive and maladaptive coping methods, following the recommendations of Meyer (2001) (36). Based on this model (Table 3), the proportion of respondents who use the various adaptive coping strategies ranged from a low of 12.9% (humor) to a high of 70.5% (acceptance); the average aggregate score was 35.7 ± 9.4. It is observed that about a fifth (20.3%) of the respondents used religion. The proportion of respondents who frequently adopt maladaptive coping, with the exception of self-distraction (33.9%), was quite low, ranging from 4.8% (substance use) to 10.4% (venting).

**Table 3:** Number (%) of respondents who frequently used the coping methods listed (*n* = 434).

**Coping methods (subscale)**	**Non-favored coping method** **(Total score: 2–5) Number (%)**	**Favored coping method** **(Total score: 6–8) Number (%)**
Active coping	235 (54.1)	199 (45.9)
Planning	320 (73.7)	114 (26.3)
Positive reframing	266 (61.3)	168 (38.7)
Acceptance	128 (29.5)	306 (70.5)
Humor	378 (87.1)	56 (12.9)
Religion	346 (79.7)	88 (20.3)
Using emotional support	338 (77.9)	96 (22.1)
Using instrumental support	341 (78.6)	93 (21.4)
Self-distraction	287 (66.1)	147 (33.9)
Denial	411 (94.7)	23 (5.3)
Venting	389 (89.6)	45 (10.4)
Substance use	413 (95.2)	21 (4.8)
Behavioral disengagement	393 (90.6)	41 (9.4)
Self-blame	400 (92.2)	34 (7.8)

Data are presented in frequency (%).

Further analysis was performed to see if there was any difference in the preference of coping methods between staff and students (**Appendix 3**). It was found that almost half the staff (49.5%) use positive reframing frequently as a means of coping compared to students (35.8%), a difference that was statistically significant (*p* = 0.016). A statistically significant difference (*p* < 0.001) was found only for the use of religion, with staff (36.3%) being more reliant on this mode of coping compared to students (15.8%). There was no significant difference between these two groups with respect to the remaining subscales.

As shown in Table 4, the aggregate scores (average) for adaptive and maladaptive coping were 35.7 (55.8%) and 19.3 (40.2%), respectively.

**Table 4:** Aggregate scores for adaptive and maladaptive coping strategies.

**Coping strategy**	**Category**	**All (*n* = 434)** **Results:** **Mean ±SD**
Adaptive (item 1–8) [Total score = 32 x 2]	Mean ± Standard deviation	35.7 ± 9.4
	Median (Interquartile range)	35.0 (13.0)
	Range	16–64
Maladaptive (item 9–14) [Total score = 24 x 2]	Mean ± Standard deviation	19.3 ± 6.2
	Median (Interquartile range)	18.0 (7.0)
	Range	12-48

### Anxiety related to the COVID-19 pandemic

The Coronavirus anxiety scale was assessed using the Cronbach's alpha reliability method; Cronbach's alpha value was 0.683 indicative of satisfactory level of reliability (**Appendix 4**). The frequency of response for each item in the Coronavirus anxiety scale is summarized in **Appendix 5**.

Table 5 is a summary of the descriptive analysis of anxiety level related to COVID-19 according to the CAS scale. As recommended, scores ranging from 0–8 are considered to indicate low level anxiety while scores of 9 or higher to indicate high level anxiety or dysfunctional anxiety. The results show that 430 respondents (99.1%) had scores ranging from 0–8, of whom 261 (60.1%) scored zero which is indicative of the absence of any symptoms of anxiety (henceforth referred to as normal). The remaining 169 (38.9%) will be considered to have mild-moderate level of anxiety. Only a very small number of the respondents (*n* = 4; 0.9%) had scores that indicate the presence of dysfunctional anxiety. There is no significant difference in the anxiety scores between staff and students.

**Table 5:** Summary of result on anxiety level related to the COVID-19 pandemic (*n* = 434).

**Assessment (minimum and maximum score)**	**Category**	**All (*n* = 434)**	**Staff (*n* =93)**	**Students (*n* = 341)**	***p*-values**
Coronavirus Anxiety Scale (0–20)	Mean ± Standard deviation	1.1 ± 1.8	0.9 ± 1.9	1.1 ± 1.8	0.324
	Median (Interquartile range)	0 (1)	0 (1)	0 (2)	-
	Range	0-11	0-10	0-11	-
	No anxiety symptoms (Score = 0)	261 (60.1)	62 (66.7)	199 (58.4)	0.100^*^
	Mild-moderate anxiety (Score =1-8)	169 (38.9)	29 (31.2)	140 (41.1)	
	Dysfunctional anxiety (Score ≥9)	4 (0.9)	2 (2.2)	2 (0.6)	

Data are presented either in mean ± standard deviation, median (interquartile range), range, or n (%).

*p-value of Fisher's Exacttest.

### Predictors of anxiety

Based on multiple linear regression analysis (Table 6), the factors associated with coronavirus anxiety score were maladaptive coping [Adjusted B coefficient (standard error) = 4.106 (0.902), *p*-value < 0.001] and presence of comorbidities [Adjusted B coefficient (Standard error) = 1.376 (0.610), *p*-value = 0.025]. The mediating effects of compliance with preventive measures, frequency of updating about the COVID-19 pandemic, self-risk perception, preparedness and perceived self-efficacy, and unwanted behavior, on the association between maladaptive coping and coronavirus anxiety were not significant (**Appendix 6**).

**Table 6:** Factors associated with Coronavirus Anxiety (*n* = 434).

	**Simple linear regression**	***p*-values**	**Multiple linear**	***P*-values**
	**Crude B coefficient (S.E.)**		**regression**	
			**Adjusted B coefficient (S.E)**	
Age	−0.009 (0.010)	0.404	−0.017 (0.020)	0.395
Gender	0.259 (0.178)	0.147	0.320 (0.175)	0.068
(Females as indicator; Males as reference)				
**Role**		0.77		
Admin vs. Academic	−0.167 (0.292)	0.568	0.131 (0.446)	0.12
Student vs Academic	0.210 (0.213)	0.324	0.728 (0.467)	
**Marital status**		0.541		
Married vs. Single	0.031 (0.281)	0.913	0.283 (0.463)	0.985
Divorced vs. Single	−1.063 (1.289)	0.41	0.024 (1.321)	
**Education level**		0.259		
Secondary vs. Postgraduate	−0.005 (0.224)	0.982	−0.629 (0.556)	0.482
Diploma or Bachelor degree vs. Postgraduate	0.373 (0.361)	0.302	0.344 (0.489)	
**Presence of comorbidities**		0.116		
Unsure vs. None	0.658 (0.397)	0.098	0.606 (0.385)	0.025
With comorbidities vs. None	1.416 (0.609)	0.021	1.376 (0.610)	0.071
Zone of residence (Green zone as indicator; Not in green zone as reference)	0.493 (0.349)	0.159	0.610 (0.337)	0.866
Household size (Staying alone as indicator; Not staying alone as reference)	−0.281 (0.494)	0.57	−0.084 (0.499)	0.598
Living with children (With children as indicator; Without children as reference)	−0.089 (0.183)	0.629	0.098 (0.186)	0.22
Living with elderly (With elderly as indicator; Without elderly as reference)	0.252 (0.187)	0.177	0.224 (0.182)	0.284
Not satisfied with support and resources	−0.504 (0.229)	0.028	0.239 (0.223)	0.692
Compliance with preventive measures	−0.064 (0.088)	0.466	−0.037 (0.093)	0.168
Frequency of updating about COVID-19	0.127 (0.062)	0.041	0.083 (0.060)	0.709
Self-risk perception	0.040 (0.033)	0.221	0.012 (0.032)	0.102
Preparedness & perceive self-efficacy	−0.053 (0.051)	0.297	0.088 (0.053)	
Unwanted behavior	0.068 (0.026)	0.009	0.049 (0.026)	0.055
Adaptive coping	0.031 (0.009)	0.001	−0.005 (0.012)	0.646
Log_10_ transformed Maladaptive coping	4.439 (0.674)	< 0.001	4.106 (0.902)	< 0.001

The adjusted R-squared values for the independent variables included in the model for multiple linear regression (enter method) for the Coronavirus Anxiety was 0.111.

## Discussion

This study aimed to determine (i) the coronavirus anxiety levels among UTAR's staff and students during the COVID-19 pandemic, (ii) the coping strategies adopted, and (iii) the predictors of coronavirus-related anxiety, adjusting for potential confounding variables.

### Coronavirus anxiety

Various studies have been carried out, mainly during the initial onslaught of the COVID-19 pandemic, to study its effects on mental health, both among the community as well as selective target populations. The reported prevalence of anxiety in the community varied from 31.9% (1) to 41.3% (37). An online survey among Malaysian university students, conducted during the first wave of the infection in early 2020 found that 20.4 percent reported minimal to moderate anxiety symptoms, while 6.6 and 2.8% reported marked to severe and extreme levels of anxiety respectively (35). In another cross-sectional survey conducted in China among college students in the midst of the outbreak in Wuhan, the reported rate of anxiety was 11.0% (38). In comparison, we found that 41% of our students experienced mild to moderate symptoms of anxiety (score of 1–8; CAS) and only 0.6% (score ≥9) had dysfunctional anxiety. The corresponding results among staff were 31.2 and 2.2% respectively.

Our study was conducted almost 1 year into the pandemic, amidst the third wave of COVID-19 in Malaysia. We found that a large proportion of the respondents in this study either reported absence of anxiety symptoms (60.1%) or mild to moderate anxiety (39.0%); only 4 individuals (0.9%) had symptoms indicative of dysfunctional anxiety. There was no significant difference between staff and students overall; however, the proportion of dysfunctional anxiety was higher among staff (2.2%) compared to students (0.6%). These results cannot be compared directly with those quoted; our study subjects are staff and students from a single privately run university in the Klang Valley in Malaysia. Secondly, the tool used for evaluation of the anxiety state is variable across studies. Further, the present study involves people who have gone through the initial wave of COVID-19 as well as several cycles of movement restrictions. Hence, the circumstances were also quite different. In addition, the university in question (UTAR) had been very proactive and had instituted relevant measures to inform, instruct, advice and support students and staff alike throughout the course of the COVID-19 outbreak. This is reflected in the high level of satisfaction with supportive measures provided. Hence, the relatively low numbers who experience dysfunctional anxiety. Nevertheless, the general consensus is that the pandemic has taken a toll on mental health and cause anxiety level to increase across all spectrums of society, albeit to different degree and extent.

### Coping strategies

The Brief COPE was used as the tool to assess the coping methods preferred by respondents of this study; from the practical point of view, this tool is simple to administer and uses a fairly standard scoring procedure; further, it is a widely validated tool with good psychometric properties. The subscales can be conveniently classified into 2 or 3 categories; in this study we divided them into adaptive and maladaptive approaches (36). The most frequent coping method used by the respondents was acceptance (70.5%), followed by active coping (45.9%) and positive reframing (38.7%), all of which are considered to be adaptive coping practices. These 3 subscales also fall into the approach coping category based on the approach-avoidant 2-factor model. Self-distraction, a maladaptive form of coping was also frequently used (33.9%); this, according to the approach-avoidant model is an avoidant behavior. We note that the common models used for classification of coping styles are somewhat over-simplistic and that overlapping classification is frequent (39).

It is noteworthy that the proportion of respondents who frequently adopt maladaptive coping, with the exception of self-distraction, was quite low, ranging from 4.8% (substance use) to 10.4% (venting). In comparison, the proportion of respondents who use the various adaptive coping methods ranged from a low of 12.9% (humor) to a high of 70.5% (acceptance). It is observed that about a fifth (20.3%) of the respondents used religion, this is apparently more so among staff than students (*p* < 0.001); likewise, the use of positive reframing, an adaptive coping method (*p* = 0.016). It is acknowledged that coping is a rather complex process that is influenced by multiple factors underlying both the situational and dispositional coping responses. In this study, the situation underlying the stress posed is the COVID-19 pandemic and all its associated negative impacts, existential, psychological, social and economic. Overall, we observe positive coping in a relatively larger proportion of the university community, which we believe is related partly to the fact that, at least among staff, a stable job with an assured income and partly to the support that staff and students received from the university throughout the entire duration of the local outbreak to date.

### The predictors of coronavirus-related anxiety

Multiple linear regressions indicated that maladaptive coping and presence of comorbidities are the significant predictors of coronavirus-related anxiety in this study. It is hypothesized that maladaptive coping strategies would lead to development of more prominent or severe pandemic-related psychological symptoms. This was confirmed in a study that demonstrated a strong association between the use of maladaptive coping strategies and anxiety symptoms in relation to the pandemic (40); in particular, self-blame was found to be related to more severe anxiety during the COVID-19 pandemic. In another study of the Australian population (41), low scores in the adaptive coping strategies, acceptance and instrumental support and high scores in the maladaptive coping strategies, behavioral disengagement and self-blame, were predictors of anxiety during COVID-19 pandemic. We did not find any significant association between adaptive coping and anxiety in this study. Some other studies have reported either the lack of or relatively weak association between adaptive coping strategies and anxiety (29–33). Interestingly, one of these studies (32) indicated that adaptive coping is associated with a higher level of subjective well-being, despite the presence of psychological disorders. However, we did not measure subjective well-being in this study, so we are unable to determine whether this is true in our case.

The finding in this study that comorbidities is a predictor of coronavirus-related anxiety is in accordance with that of a previous study (42) which found that people with underlying comorbidities are more likely to have high anxiety score than those without. This is not unexpected as it is widely known and accepted that people with comorbidities are at higher risk of morbidity and mortality from COVID-19; hence, the increased anxiety among this group of people (43).

Lastly, the majority of respondents indicated that that were compliant with recommended public health measures, and were satisfied with the support and resources provided by the university during the pandemic. It might be surmised that this behavior and the satisfaction with support services are contributory to the very low frequency of dysfunctional anxiety and the relatively low frequency of anxiety symptoms among the respondents, staff and student alike. About one-tenth of respondents update themselves about the status of the COVID-19 pandemic several times a day, which could contribute to heightened anxiety reported by some respondents.

### Implications

This study informs about the effect of the COVID-19 pandemic on the mental status of staff and students of a university community, and its association with the coping methods employed. To this end, we employed the Coronavirus Anxiety Scale, a validated instrument to explore the anxiety level, and the Brief COPE to examine the coping styles favored among staff and students in the midst of the pandemic.

### Limitations

This study is an observational study confined to a single university community, which limits the generalization of the results to other educational institutes. Secondly, participants might not be truly representative of the university community as a whole as they were recruited *via* an online survey using universal sampling method. The Coronavirus anxiety scale used is based on reported symptoms of anxiety and provided only 2 classifications—mild anxiety (score 0–8) and dysfunctional anxiety (score ≥9). The factors associated with anxiety were not examined in detail as the number of subjects who reported severe or dysfunctional anxiety was too few.

Further, it is acknowledged that the Brief-COPE instrument has not been adequately validated in the Malaysian population and so it is not known how well the latent constructs of adaptive and maladaptive coping strategies translate to actual coping ability. Therefore, further validation studies with larger sample size and representative sampling methods are warranted, to allow for more in-depth appropriate and comprehensive analyses that may verify these findings.

We are also cognisant of the fact that during the pandemic, travel and social activities were extremely restricted. It is likely that many would spend more time on smartphones and the internet; appropriate use of smartphones and the internet could provide the means for handling the distress due to these restrictions (44). However, inappropriate use or overuse of the smartphone and internet, in particular to the extent of addiction could conversely exacerbate distress and coronavirus-related anxiety (45). However, we did not capture the pattern of smartphone and internet use in this study; therefore, the results on this aspect should be interpreted with this limitation in view.

## Conclusion

A high score for maladaptive coping and presence of comorbidities were the predictors of coronavirus anxiety. Dysfunctional anxiety among the UTAR community, a not-for-profit private university situated in the Klang Valley, Malaysia is very low at < 1 percent. This is believed to be reflective of the high level of satisfaction with the support and services provided during the COVID-19 outbreak. With respect to coping methods employed during the outbreak, it was found that adaptive coping methods were used a lot more frequently by both staff and students. Nevertheless, the study has identified small numbers of people who practice maladaptive coping behavior which can act as prompts for appropriate action/intervention.

## Data availability statement

The raw data supporting the conclusions of this article will be made available by the authors, without undue reservation.

## Ethics statement

The studies involving human participants were reviewed and approved by Universiti Tunku Abdul Rahman's Scientific and Ethical Review Committee. Ethics approval number: U/SERC/138/2020. The patients/participants provided their written informed consent to participate in this study.

## Author contributions

Conceptualization and methodology: KWL, SFY, HTO, and MSL. Data curation: MSL. Formal analysis and writing—original draft: KWL, SFY, and MSL. Funding acquisition: SFY. Investigation: KWL, SFY, HTO, KSP, and MSL. Project administration and resources: HTO. Software: KWL and KSP. Supervision: SFY and MSL. Validation: KSP and MSL. Visualization: KWL. Writing—review & editing: KWL, SFY, KSP, and MSL.

## Funding

This research was supported by Universiti Tunku Abdul Rahman (Grant Number: IPSR/RMC/UTARRF/2020-C2/Y01).

## Conflict of interest

The authors declare that the research was conducted in the absence of any commercial or financial relationships that could be construed as a potential conflict of interest.

## Publisher's note

All claims expressed in this article are solely those of the authors and do not necessarily represent those of their affiliated organizations, or those of the publisher, the editors and the reviewers. Any product that may be evaluated in this article, or claim that may be made by its manufacturer, is not guaranteed or endorsed by the publisher.
