# Supplementary material for: Anxiety and coping strategies during the COVID-19 pandemic: A cross-sectional study of staff and students from a tertiary education center in Malaysia
Source: Front Public Health. 2022 Oct 6;10:936486. doi: 10.3389/fpubh.2022.936486 (PMC9583838; doi:10.3389/fpubh.2022.936486)
Supplement: Supplementary file 1 [file Data_Sheet_1.PDF]

**Appendix 1** Internal consistency of Brief-COPE				
Coping mechanism	Coping subscales	Item number in Brief-COPE	Cronbach's alpha coefficients of original scale	Cronbach's alpha coefficients in current study
Adaptive coping	Active coping	2 and 7	0.680	0.630
	Planning	14 and 25	0.730	0.576
	Positive reframing	12 and 17	0.640	0.685
	Acceptance	20 and 24	0.570	0.796
	Humor	18 and 28	0.730	0.852
	Religion	22 and 27	0.820	0.791
	Using emotional support	5 and 15	0.710	0.659
	Using instrumental support	10 and 23	0.640	0.761
Maladaptive approach	Self-distraction	1 and 19	0.710	0.590
	Denial	3 and 8	0.540	0.775
	Venting	9 and 21	0.500	0.603
	Substance use	4 and 11	0.900	0.936
	Behavioural disengagement	6 and 16	0.650	0.724
	Self-blame	13 and 26	0.690	0.744

**Appendix 2** Assessment of Coping style using Brief COPE among respondents (n = 434)				
Brief COPE Items	I haven't been doing this at all (score 1)	I've been doing this a little bit (score 2)	I've been doing a medium amount (score 3)	I've been doing this a lot (score 4)
1. I've been turning to work or other activities to take my mind off things.	94 (21.7)	140 (32.3)	133 (30.6)	67 (15.4)
2. I've been concentrating my efforts on doing something about the situation I'm in.	46 (10.6)	172 (39.6)	116 (26.7)	100 (23.0)
3. I've been saying to myself "this isn't real".	332 (76.5)	28 (6.5)	59 (13.6)	15 (3.5)
4. I've been using alcohol or other drugs to make myself feel better.	393 (90.6)	14 (3.2)	15 (3.5)	12 (2.8)
5. I've been getting emotional support from others.	217 (50.0)	82 (18.9)	101 (23.3)	34 (7.8)
6. I've been giving up trying to deal with it.	287 (66.1)	46 (10.6)	84 (19.4)	17 (3.9)
7. I've been taking action to try to make the situation better.	74 (17.1)	142 (32.7)	141 (32.5)	77 (17.7)
8. I've been refusing to believe that it has happened.	344 (79.3)	22 (5.1)	61 (14.1)	7 (1.6)
9. I've been saying things to let my unpleasant feelings escape.	254 (58.5)	39 (9.0)	116 (26.7)	25 (5.8)
10. I've been getting help and advice from other people.	197 (45.4)	77 (17.7)	124 (28.6)	36 (8.3)
11. I've been using alcohol or other drugs to help me get through it.	389 (89.6)	11 (2.5)	24 (5.5)	10 (2.3)
12. I've been trying to see it in a different light, to make it seem more positive.	134 (30.9)	132 (30.4)	106 (24.4)	62 (14.3)
13. I've been criticizing myself.	282 (65.0)	40 (9.2)	90 (20.7)	22 (5.1)
14. I've been trying to come up with a strategy about what to do.	109 (25.1)	134 (30.9)	142 (32.7)	49 (11.3)
15. I've been getting comfort and understanding from someone.	140 (32.3)	131 (30.2)	116 (26.7)	47 (10.8)
16. I've been giving up the attempt to cope.	286 (65.9)	45 (10.4)	80 (18.4)	23 (5.3)
17. I've been looking for something good in what is happening.	82 (18.9)	147 (33.9)	113 (26.0)	92 (21.2)
18. I've been making jokes about it.	229 (52.8)	48 (11.1)	127 (29.3)	30 (6.9)
19. I've been doing something to think about it less, such as going to movies, watching TV, reading, daydreaming, sleeping, or shopping.	117 (27.0)	125 (28.8)	120 (27.6)	72 (16.6)
20. I've been accepting the reality of the fact that it has happened.	43 (9.9)	131 (30.2)	53 (12.2)	207 (47.7)
21. I've been expressing my negative feelings.	196 (45.2)	73 (16.8)	140 (32.3)	25 (5.8)

22. I've been trying to find comfort in my religion or spiritual beliefs	186 (42.9)	74 (17.1)	126 (29.0)	48 (11.1)
23. I've been trying to get advice or help from other people about what to do.	178 (41.0)	100 (23.0)	121 (27.9)	35 (8.1)
24. I've been learning to live with it.	37 (8.5)	171 (39.4)	84 (19.4)	142 (32.7)
25. I've been thinking hard about what steps to take.	128 (29.5)	94 (21.7)	157 (36.2)	55 (12.7)
26. I've been blaming myself for things that happened.	324 (74.7)	35 (8.1)	55 (12.7)	20 (4.6)
27. I've been praying or meditating.	199 (45.9)	64 (14.7)	128 (29.5)	43 (9.9)
28. I've been making fun of the situation.	285 (65.7)	39 (9.0)	93 (21.4)	17 (3.9)
Data are presented in frequency (%).				

Appendix 3 Comparison of coping strategies between **staff and students** (n = 434)

Coping Style	Coping subscales		Subscale Scores		p-values
			Staff (n=93)	Students (n=341)	
Adaptive	Active coping	Less frequently used	49 (52.7)	186 (54.5)	0.750
		More frequently used	44 (47.3)	155 (45.5)	
	Planning	Less frequently used	66 (71.0)	254 (74.5)	0.494
		More frequently used	27 (29.0)	87 (25.5)	
	Positive reframing	Less frequently used	47 (50.5)	219 (64.2)	**0.016**
		More frequently used	46 (49.5)	122 (35.8)	
	Acceptance	Less frequently used	28 (30.1)	100 (29.3)	0.883
		More frequently used	65 (69.9)	241 (70.7)	
	Humor	Less frequently used	85 (91.4)	293 (85.9)	0.163
		More frequently used	8 (8.6)	48 (14.1)	
	Religion	Less frequently used	59 (63.4)	287 (84.2)	**<0.001**
		More frequently used	34 (36.6)	54 (15.8)	
	Emotional support	Less frequently used	74 (79.6)	264 (77.4)	0.658
		More frequently used	19 (20.4)	77 (22.6)	
	Instrumental support	Less frequently used	69 (74.2)	272 (79.8)	0.246
		More frequently used	24 (25.8)	69 (20.2)	
Maladaptive	Self-distraction	Less frequently used	57 (61.3)	230 (67.4)	0.266
		More frequently used	36 (38.7)	111 (32.6)	
	Denial	Less frequently used	88 (94.6)	323 (94.7)	1.000
		More frequently used	5 (5.4)	18 (5.3)	
	Venting	Less frequently used	83 (89.2)	306 (89.7)	0.891
		More frequently used	10 (10.8)	35 (10.3)	
	Substance use	Less frequently used	89 (95.7)	324 (95.0)	1.000
		More frequently used	4 (4.3)	17 (5.0)	
	Behavioural disengagement	Less frequently used	82 (88.2)	311 (91.2)	0.376
		More frequently used	11 (11.8)	30 (8.8)	
	Self-blame	Less frequently used	97 (93.5)	313 (91.8)	0.576
		More frequently used	6 (6.5)	28 (8.2)	
Note: *Total subscale score: 2 to 5 = less frequently used; 6 to 8 = more frequently used.					

**Appendix 4** Reliability analysis for Coronavirus Anxiety Scale				
Item	Scale mean if item deleted	Scale variance if item deleted	Correlated item - total correlation	Cronbach's Alpha if item deleted
1. I felt dizzy, lightheaded, or faint, when I read or listened to news about the coronavirus	0.7304	2.008	0.368	0.692
2. I had trouble falling or staying asleep because I was thinking about the coronavirus	0.8571	2.317	0.474	0.618
3. I felt paralyzed or frozen when I thought about or was exposed to information about the coronavirus	0.7673	2.027	0.488	0.610
4. I lost interest in eating when I thought about or was exposed to information about the coronavirus	0.9194	2.458	0.504	0.617
5. I felt nauseous or had stomach problems when I thought about or was exposed to information about the coronavirus	0.9562	2.610	0.491	0.632
Note: Reliability statistics indicated that the Cronbach's alpha value for Coronavirus Anxiety scale - 5 items was **0.683** (Alpha values of 0.65-0.95 is satisfactory, <0.65 shows that the ability of the items in the research instrument to measure the concept is low; >0.95 means all items are similar or overlap with one another, and the use of items which overlap is not encouraged.)				

**Appendix 5** Mental health screening using the Coronavirus Anxiety Scale among respondents (n = 434)					
How often have you experienced the following statements over the last 2 weeks?	Not at all	Rarely	Several days	More than 7 days	Nearly every day
1. I felt dizzy, lightheaded, or faint, when I read or listened to news about the coronavirus	337 (77.6)	68 (15.7)	20 (4.6)	2 (0.5)	7 (1.6)
2. I had trouble falling or staying asleep because I was thinking about the coronavirus	364 (83.9)	56 (12.9)	12 (2.8)	1 (0.2)	1 (0.2)
3. I felt paralyzed or frozen when I thought about or was exposed to information about the coronavirus	337 (77.6)	77 (17.7)	15 (3.5)	1 (0.2)	4 (0.9)
4. I lost interest in eating when I thought about or was exposed to information about the coronavirus	383 (88.2)	45 (10.4)	4 (0.9)	1 (0.2)	1 (0.2)
5. I felt nauseous or had stomach problems when I thought about or was exposed to information about the coronavirus	397 (91.5)	31 (7.1)	5 (1.2)	1 (0.2)	0 (0.0)
Data are presented in frequency (%).					

Appendix 6 Summary of mediating effects (results shown as indirect effects of x on y) of compliance with preventive measures, frequency of updating about the COVID-19 pandemic, self-risk perception, preparedness and perceived self-efficacy, and unwanted behaviour, on the association between maladaptive coping and coronavirus anxiety using Model 4 in Process Macro by 3 different approaches.

Potential mediators	Approach 1: Five mediators were analysed separately				Approach 2: Five mediators were analysed in parallel without covariates				Approach 3: Five mediators were analysed in parallel with 15 covariates			
	**Effect**	**Boot SE**	**Boot LLCI**	**Boot ULCI**	**Effect**	**Boot SE**	**Boot LLCI**	**Boot ULCI**	**Effect**	**Boot SE**	**Boot LLCI**	**Boot ULCI**
Compliance with preventive measures	.0065	.0416	-.0646	.1154	.0025	.0364	-.0589	.0999	.0320	.0971	-.1439	.2594
Frequency of updating about the COVID-19 pandemic	.1075	.0955	-.0422	.3350	.1178	.1011	-.0392	.3543	.0514	.0858	-.1111	.2461
Self-risk perception	.0554	.0856	-.0724	.2701	.0379	.0742	-.0684	.2391	.0344	.0912	-.0911	.2792
Preparedness and Self-efficacy	-.0577	.0694	-.2212	.0530	-.0476	.0729	-.2370	.0598	.0340	.0925	-.1347	.2453
Unwanted behaviour	.1878	.1698	-.0869	.5811	.2038	.1684	-.0773	.5862	.2331	.1917	-.0740	.6619
